# Optimal surgical margin for penile‐sparing surgery in management of penile cancer—Is 2 cm really necessary?

**DOI:** 10.1002/bco2.75

**Published:** 2021-02-05

**Authors:** Elliot Anderson, Henry Han‐I Yao, Justin Chee

**Affiliations:** ^1^ Department of Urology Western Health Melbourne VIC Australia; ^2^ Eastern Health Clinical School Monash University Melbourne VIC Australia; ^3^ Department of Urology Alfred Health Melbourne VIC Australia

**Keywords:** glansectomy, partial penectomy, penile cancer, penile‐sparing therapy, surgical margin

## Abstract

**Introduction:**

Classical teaching of a 2 cm macroscopic surgical margin for surgical treatment of primary penile cancer is overly aggressive. Contemporary evidence suggests narrow but clear margins have similar survival outcomes for localized disease. This study aims to determine the oncological outcome of using a risk‐adapted algorithm to selection of macroscopic surgical margin based on biopsy grade of disease: 5 mm margin for grade 1, 10 mm margin for grade 2, and 20 mm margin for grade 3.

**Methods:**

This is a retrospective case series of patients who underwent penile‐sparing surgery for biopsy‐proven penile SCC by a single surgeon from May 2010 through to January 2019. Clinicopathological data were extracted from medical records. Primary outcome was the positive margin rate. Secondary outcomes were overall survival (OS), cancer‐specific survival (CSS), metastasis‐free survival (MFS), and local recurrence‐free survival (RFS). Kaplan‐Meier survival analysis was used to determine survival outcomes.

**Results:**

A total of 21 patients were included in this study. The median age was 65. Pre‐operative biopsy grade was grade 1 in 19.1% of patients, grade 2 in 47.6%, and grade 3 in 33.3%. The median size of tumor on examination was 20 mm. Using a grade‐stratified algorithm for macroscopic surgical margin, only one patient (4.8%) had a positive margin. This patient had G1T3 disease and proceeded to have a total penectomy for oncological clearance. The median margin clearance was 7 mm. The 12‐month OS, CSS, MFS, and local RFS were 94.6%, 94.6%, 81.0%, and 92.3%, respectively.

**Conclusion:**

This study suggests that using a grade‐stratified approach to aim for a narrower macroscopic surgical margin does not appear to significantly alter the oncological outcome, with a negative margin rate of 95.2% in our this series. This enables more men to be eligible for organ preserving surgery and thereby improve their quality of life in the urinary function and sexual function domain. Larger prospective studies are warranted to confirm these findings.

## 1 INTRODUCTION

Penile cancer is a rare form of cancer with an incidence of 0.66‐1.44 per 100 000 men.^1^, ^2^, ^3^ The majority of penile cancer can be histologically classified as squamous cell carcinoma.^2^ The goal of local management for penile cancer is to achieve complete tumor removal as the most important priority followed by organ preservation to achieve a better functional and cosmetic outcome.^4^, ^5^ The majority of penile cancer are located on the glans or foreskin, making them amenable to organ‐sparing surgery.^2^

Traditional teaching recommends a 2 cm macroscopic surgical margin for all tumors, which significantly limits the number of men suitable for penile‐sparing surgery.^6^, ^7^, ^8^ Hoffman et al. was the first to propose that local oncological control can be obtained with margins less than 15 mm.^9^ More contemporary case series of partial and total penectomy have demonstrated that none of the grade 1 or 2 lesions microscopically extended beyond 10 mm proximal to the visible margin, and none of the grade 3 lesions extended beyond 15 mm.^10^ Furthermore, Phillipou et al found that the extent of microscopic clear excision margin of 5 mm or less versus greater than 5 mm was not an independent predictor of local recurrence.^11^ With the use of intra‐operative frozen section and ability to re‐operate in the event of positive margin, we can be less aggressive with our macroscopic margin and not subject all patients to a 2 cm macroscopic surgical clearance.^12^ This will allow more patients to be suitable for penile‐sparing surgery leading to a better functional outcome and quality of life. This study used a risk‐adapted approach to the selection of macroscopic surgical margin to achieve based on pre‐operative biopsy tumor grade: 5 mm macroscopic surgical margin in grade 1 disease, 10 mm margin in grade 2 disease, and 20 mm margin in grade 3 disease. This study aims to assess the oncological outcomes of this risk‐adapted algorithm to the selection of macroscopic surgical margin.

## 2 METHODS

This is a retrospective single surgeon case series. From January 2009 to January 2019, all patients who underwent penile‐sparing surgery for management of biopsy‐proven penile squamous cell carcinoma were included in this study. All cases were performed by a single surgeon who is the sole provider of penile cancer surgery at the included institutions. All patients underwent biopsy of the primary tumor prior to definitive surgery. Biopsy was either performed in the office setting or in theatre for patients who choose to be under general anesthesia for their biopsy. This study used a risk‐adapted algorithm based on biopsy tumor grade to determine the macroscopic surgical margin to achieve at the time of operation. Surgical resection margin was planned with the aid of optical loupes and a sterile ruler, and the macroscopic surgical margin achieved was 5 mm for grade 1 disease, 10 mm for grade 2 disease, and 20 mm for grade 3 disease. Clinicopathological data were extracted from medical records and included: age, macroscopic tumor size, biopsy tumor grade, surgical management, macroscopic margin, length of stay, complications, histological tumor size, histological tumor grade, histological lymphovascular invasion, pathological stage, surgical margin status, lymph node management, chemotherapy given, urethral stricture on follow‐up, voiding satisfaction, incontinence, local recurrences, metastasis and survival status. Tumor stage was categorized according to the 8th edition of the Tumor Node Metastasis (TNM) classification on cancer staging developed by the American Joint Commission on Cancer (AJCC).^13^

Primary outcome of this study was the positive surgical margin rate. Secondary outcomes of this study were overall survival (OS), cancer‐specific survival (CSS), metastasis‐free survival (MFS), and local recurrence‐free survival (RFS). Statistical analyses were performed using *Stata Statistical Software: Release 16* (StataCorp LLC, Texas, United States). Kaplan‐Meier survival analyses were performed for survival outcomes. Univariate analyses using log‐rank test were used to compare factors that may predict survival outcomes. Multivariate analysis using cox proportional hazard model was not able to be performed due to the small numbers in this study.

Ethics approval (WH‐55,113, AH‐593/20) was obtained from participating institutions. This study conformed to the provisions of the Declaration of Helsinki.^14^

## 3 RESULTS

Twenty‐one patients were included in this study. The median age was 65 (IQR 49‐74). The median macroscopic tumor size was 20 mm (IQR 20‐35). Preoperative biopsy grade was grade 1 in 19.1% (n = 4) of patients, grade 2 in 47.6% (n = 10), and grade 3 in 33.3% (n = 7). The breakdown of surgical management, lymph node management, and chemotherapy are listed in Table 1. The median length of stay was 2 days (IQR 1.5‐3). The median histological tumor size was 35 mm (IQR 20‐40). Histopathological tumor grade was CIS only in 9.5% (n = 2) of patients, grade 1 in 14.3% (n = 3), grade 2 in 38.1% (n = 8), and grade 3 in 38.1% (n = 8). Two patients (9.5%) had upgrading of tumor grade from biopsy to final histopathology and two patients (9.5%) had downgrading of tumor grade (Table 2). Overall, 81% of pathology were concordant between biopsy and final histopathological tumor grade, and 100% concordant for those with grade 3 disease on biopsy (n = 7). Pathological T stage was reported to be Tis in 10.5% (n = 2) of patients, T1a in 26.3% (n = 5), T1b in 15.8% (n = 3), T2 in 36.8% (n = 7), and T3 in 10.5% (n = 2) patients. Four patients were found to have nodal disease in the inguinal nodes.

**TABLE 1:** Breakdown of local surgical management, lymph node management, and chemotherapy treatment for patients in this study

	Number	Percentage (%)
Surgical management		
Circumcision	1	4.8
Wide local excision	1	4.8
Glansectomy	3	14.3
Partial penectomy	13	61.9
Penectomy	3	14.3
Lymph node management		
Bilateral radical ILND	4	19.1
Bilateral SLNB	9	42.9
Left excisional LN biopsy	1	4.8
Right excisional LN biopsy	1	4.8
Right excisional LN biopsy and left SLNB	1	4.8
Left SLNB and right superficial ILND	2	9.5
Left superficial ILND and right SLNB	1	4.8
None	2	9.5
Neoadjuvant chemotherapy		
Yes	1	4.8
No	20	95.2
Adjuvant chemotherapy		
Yes	3	14.3
No	18	85.7

**TABLE 2:** Discrepancy between biopsy tumor grade and final histopathological tumor grade

Biopsy tumor grade	Final histopathology tumor grade				
	CIS	1	2	3	Total
1	0	3	1	0	4
2	2	0	7	1	10
3	0	0	0	7	7
Total	2	3	8	8	21

Using this study's risk‐adapted algorithm for macroscopic surgical margin, positive surgical margin was present in only one patient (4.8%). This patient had biopsy diagnosed grade 1 disease but was found to have T3 cancer on his partial penectomy histopathology. He subsequently underwent a total penectomy to achieve local clearance of tumor. He did not have any further recurrences or metastasis and was alive at last follow‐up of 94 months. The median microscopic margin clearance in this study was 7 mm (IQR 2‐10).

Over the study period, two patients developed local recurrence. The first patient had recurrent CIS that was excised. The second patient had a grade 2 SCC recurrence that was treated with WLE. This recurred a second time and the patient proceeded to a partial penectomy. Four patients developed metastatic SCC on follow‐up. Five patients died during follow‐up of which four died from metastatic SCC. All four patients with metastatic SCC had clear margins on primary tumor excision and did not exhibit any local recurrence on follow‐up. Of these patients, three had positive lymph nodes on lymph node dissection and one did not undergo lymph node clearance.

On univariate analysis using log‐rank test macroscopic surgical margin (*P* = 0.19), lymphovascular invasion (*P* = 0.19), and pathological T stage (*P* = 0.38) were not predictors for CSS. Histological grade (*P* = 0.035) and presence of nodal disease (*P* = 0.0005) were predictors of CSS on univariate analysis using log‐rank test.

**FIGURE 1:**
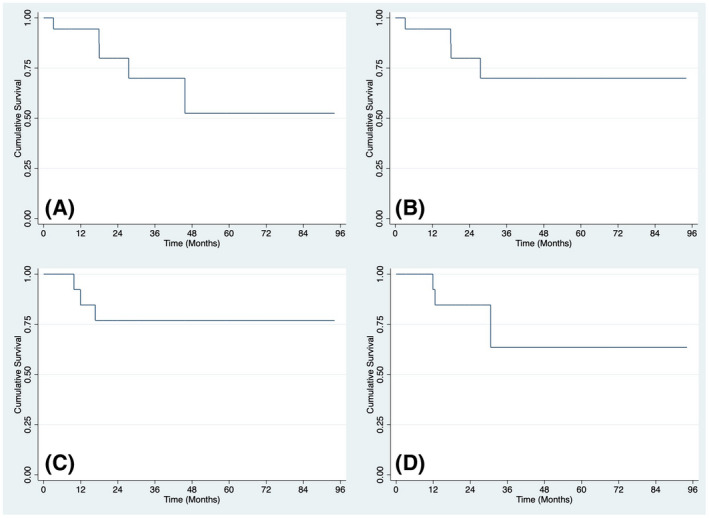
Survival curves for men who underwent penile‐sparing surgery in this case series. (A) Overall survival, (B) Cancer‐specific survival, (C) Metastasis‐free survival, and (D) Local recurrence‐free survival

The median follow‐up for OS and CSS was 19.3 months (IQR 8.2‐30.6). The median follow‐up for MFS was 16.7 months and for local RFS was 17.9 months. The 12 and 24 months OS were 94.6% and 80.0%, respectively (Figure 1). The 12 and 24 months CSS were 94.6% and 80.0%, respectively (Figure 1). The 12 and 24 months MFS were 81.0% and 73.6%, respectively (Figure 1). The 12 and 24 months local RFS were 92.3% and 84.6%, respectively (Figure 1).

Two complications (9.5%) were reported in this study. One patient presented with a blocked urethral catheter, this was unblocked in the emergency department, and the patient discharged home. The second patient developed a penile abscess following a glansectomy that required return to theatre for debridement.

Of those patients with clinical follow‐up of functional outcome, no patient developed urethral strictures (n = 0/15) or incontinence (n = 0/11), and 91.7% (n = 11/12) reported to be satisfied with their voiding function.

## 4 DISCUSSION

Currently, there is no clear evidence regarding the optimal width of clear surgical margin for best oncological outcome in penile cancer surgery.^15^ Conventionally, a 2 cm surgical margin is advocated for all penile cancers, which limits the number of men suitable for organ‐sparing surgery.^6^, ^7^, ^8^ A study has shown that the extent of microscopic surgical margin of 5 mm or less versus greater than 5 mm was not a predictor of local recurrence.^11^ Therefore, achieving a clear surgical margin even if less than 5 mm does not compromise local control.^11^ Another study, by Sri et al, further suggested that a deep clear margin of >1 mm is adequate and has a low risk of local recurrence.^16^ As such EAU guidelines have recommended that a 3‐5 mm width of negative histopathological margin is adequate.^15^ Although the EAU guideline did comment on the use of a grade‐based approach to determine the width of negative surgical margin to achieve, it was unclear whether this referred to macroscopic margin to aim for or microscopic margin identified on histology. Furthermore, this recommendation is based on expert opinion of the panel. This study supports the expert opinion, demonstrating that with a risk‐adapted approach to the selection of target macroscopic resection margin, the risk of positive margin is low. For these small percentage of patients, they can undergo further resection to achieve negative surgical margin.^11^ Even in the event of local recurrence, CSS does not appear to be adversely affected.^11^, ^15^, ^17^ Studies have found favorable 5‐year CSS rates of 91.7%‐92% even for patients with local recurrence.^11^, ^17^ However, a recent large retrospective multicenter study has challenged these studies and has shown local recurrence to be a significant predictor of decreased OS and CSS on multivariate analysis.^18^ Nodal disease^18^ and regional recurrence are more well‐established determinants of poor CSS, with a 5‐year CSS of 32.7%‐38.4% in patients with regional recurrence.^11^, ^17^ These findings suggest that mortality relating to penile cancer is more closely related to nodal and metastatic disease than local recurrence. Therefore, it is unnecessary to achieve a 2 cm macroscopic resection margin for all patients. A risk‐adapted algorithm like the one used in this study would allow more men to be eligible for organ‐sparing treatment without compromising oncological outcome.

Organ preservation surgery is associated with better functional and psychological outcomes compared with more mutilating resections.^19^ Keiffer et al found that men who underwent partial penectomy compared with penile‐sparing surgery had more problems with orgasm, appearance concerns, life interference, and urinary function,^20^ and only 10%‐20% of men post total penectomy engage in any form of sexual activity.^21^ Significant psychological harm also appears to be associated with more aggressive resections.^22^ Ficarra et al found that following partial penectomy, men had a measurable impairment to their psychological well‐being compared to control group.^23^ In addition, a prospective Chinese study found that following a partial penectomy, 39% of men had depression and 58% suffered from anxiety.^24^ These findings highlight the importance of offering the least destructive penile‐sparing surgery that does not compromise oncological outcome.

There are several limitations to this study. Firstly, this is a retrospective study and has all the associated limitations with this design. For example, it relies on the accuracy of medical records and inevitably there will be missing data points. Importantly, the data required to determine primary and secondary outcomes of this study were all complete. However, the data for functional outcomes are incomplete, and this would be better assessed in prospective studies using patient‐reported outcome questionnaires. The second limitation of this study is its small number of cases. Given the rarity of penile cancer, it is difficult to obtain a large population for any case series. As such, due to the small numbers only univariate analyses on the predictors of cancer‐specific mortality were able to be performed and even this analysis is likely underpowered. For the purpose of this study, it does support that the macroscopic surgical margin aimed for in this study did not appear to have an impact on cancer‐specific mortality, and that histopathological grade and nodal disease are more significant factors in predicting CSS than margin size. More robust analysis into factors predicting CSS on multivariate analyses has been published by Roussel et al., and lymphovascular invasion, local recurrence, and nodal disease are all independent predictors for CSS.^18^ The findings of this study will add to the weight of the existing literature on surgical margins in penile‐sparing surgery, and support the grade‐based approach to selection of surgical resection margin suggested by the EAU guidelines.^15^

## 5 CONCLUSION

Conventional teaching of a 20 mm macroscopic resection margin for all penile cancers is likely over‐aggressive. This study suggests that a risk‐adapted algorithm to macroscopic resection margin based on biopsy tumor grade can achieve a low positive surgical margin rate, which can be further surgically treated to achieve a clear margin. The algorithm suggested in this study of aiming for a macroscopic resection margin of 5 mm for grade 1 disease, 10 mm for grade 2 disease, and 20 mm for grade 3 disease may provide a good balance between achieving a good oncological outcome and functional outcome from organ preservation. The findings of this study should be confirmed with larger prospective randomized trials with comparative groups.

## CONFLICT OF INTEREST

None.
